# Assessment of Bioceramic Sealer Retreatability and Its Influence on Force and Torque Generation

**DOI:** 10.3390/ma15093316

**Published:** 2022-05-05

**Authors:** Ahmed Jamleh, Mohannad Nassar, Abdulmohsen Alfadley, Azhar Alanazi, Hadeel Alotiabi, Maryam Alghilan, Khalid Alfouzan

**Affiliations:** 1 Restorative and Prosthetic Dental Sciences, College of Dentistry, King Saud bin Abdulaziz University for Health Sciences, National Guard Health Affairs, Riyadh 11426, Saudi Arabia; alfadley86@gmail.com (A.A.); a.s.r1416@gmail.com (A.A.); hadeel_munif@hotmail.com (H.A.); m.alghilan@hotmail.com (M.A.); kalfouzan@yahoo.com (K.A.); 2 King Abdullah International Medical Research Centre, National Guard Health Affairs, Riyadh 11426, Saudi Arabia; 3 Department of Preventive and Restorative Dentistry, College of Dental Medicine, University of Sharjah, Sharjah 27272, United Arab Emirates; minassar@sharjah.ac.ae

**Keywords:** AH Plus, MicroCT, oval canal, retreatability, TotalFill bioceramic

## Abstract

This study assesses the retreatability of TotalFill bioceramic (TFBC) and AH Plus (AHP) sealers and their impact on retreatment force and torque. Twenty-six premolar teeth with single oval canals were instrumented, obturated using the matched gutta-percha cone technique with one of the tested sealers, and then temporized. After a 6-month incubation at 37 °C and 100% humidity, the canals were retreated with the XP Shaper system. During retreatment, the generated force and torque were measured. Micro-CT scanning was run before and after the retreatment procedure to analyze the remaining obturating materials in the canals. The apically directed maximum force in AHP was higher than that in TFBC. The coronally directed maximum force and the maximum torque were comparable between the groups. A higher amount of remaining obturating materials was found in the AHP compared to that in the TFBC. Based on these findings, endodontic sealer had an influence on retreatability, and the TFBC showed less remaining obturating materials and lower retreatment forces in the apical direction compared to the AHP in extracted teeth with oval canals.

## 1. Introduction

Although root canal treatment has a high success rate [1], failure might occur even in well-treated teeth due to several microbial or non-microbial reasons [2], as evidenced by the high prevalence of periradicular radiolucencies in root canal obturated teeth [3]. This calls for retreatment that requires the removal of old obturating materials.

Endodontic nonsurgical retreatment is a comprehensive field with efforts to provide a potential space for root canal system disinfection and re-obturation [4]. This option is considered successful through addressing defects that are already present as pathologic or iatrogenic in origin and avoiding their occurrence during the procedure. Moreover, retreatment difficulties might be shown in coronal restoration disassembly, allocating missed canals, removing obturating materials, negotiating ledges and canal blockages, managing intracanal transportations, repairing root perforations, and removing endodontic posts and separated instruments [4,5,6].

Gutta-percha is the core material of choice for root canal obturation and is placed along with a biocompatible endodontic sealer. Endodontic sealers can be classified based on their chemical formulation. AH Plus sealer (AHP) (Dentsply, Tulsa, OK, USA) is an epoxy resin-based sealer that is commonly used due to its good sealing and bonding abilities between dentin and gutta-percha [7]. TotalFill bioceramic sealer (TFBC) (FKG Dentaire SA, La Chaux-de-Fonds, Switzerland) is a premixed calcium silicate-based bioceramic sealer. It is also distributed under the brand names EndoSequence BC Sealer (BUSA, Savannah, GA, USA), and iRoot SP (Innovative BioCeramix, Vancouver, BC, Canada). The sealing ability of bioceramic sealer with gutta-percha in a matched cone obturation technique was shown to be as effective as AHP [8].

The type of endodontic sealer used is one of the factors that influences retreatability of the root canal system [9,10,11,12,13,14,15,16,17,18,19]. Moreover, the literature is now very rich with reports discussing the vertical force and torque induced during initial root canal treatment [20,21,22,23]. However, no study has addressed the effect of different endodontic sealers on the vertical force and torque generated during retreatment. It was reported that the incidence of fracture in NiTi instruments used for root canal retreatment is increased by 400% compared to initial treatment [24], as instruments face several intracanal obstacles during retreatment that can impose more stress on the instruments and ultimately the root canal walls. Thus, the purpose of this study is to determine the effect of TFBC on the retreatability of obturated root canals and its impact on the generated force and torque compared with the AHP. The null hypothesis is that there is no difference between AHP and TFBC in terms of the percentage of remaining obturating materials and developed retreatment force and torque.

## 2. Materials and Methods

### 2.1. Teeth Selection

The study protocol was approved by an Ethical Committee Board from King Abdullah International Medical Research Center (RC20/570/R) on the 4th of November 2020. All methods were performed in accordance with the relevant guidelines and regulations [25]. Permanent premolar teeth with single oval canals in which the buccolingual diameter of the canal was more than two times greater than the mesiodistal diameter throughout the coronal two-thirds were chosen from a pool of human teeth extracted for unknown reasons and stored in distilled water. Informed consent was obtained before tooth extraction. The teeth were disinfected as per CDC guidelines to prevent cross contamination. They were radiographically and visually observed under a dental operating microscope (4× magnification, OPMI Zeiss Pico; Carl Zeiss MediTec, Dublin, CA, USA). Teeth with curved canals, incomplete/resorbed roots, root canal filling, or cracks were excluded. Using the two-sample t-test (PiFace, http://homepage.stat.uiowa.edu/~rlenth/Power/ (accessed on 21 October 2020)), the sample size was calculated with a power of 80% and a significance level of 5% based on preliminary data obtained from 4 samples in each group, where the minimum mean differences (common standard deviations) of the effective retreatment time, retreatment force, retreatment torque, and remaining obturating materials were found to be 9 (6), 0.5 (0.4), 0.4 (0.3), and 7 (5), respectively. Based on these calculations, the sample size attained should be at least 11 in each group. Thus, twenty-six teeth were included.

### 2.2. Sample Preparation

Each tooth root was covered with a single layer of aluminum foil and embedded in a mixed autopolymerizing resin (Duralay; Reliance Dental Mfg, Worth, IL, USA) surrounded by a plastic tube (12 mm high). After the resin set, the foil was replaced with a light-body silicone impression material to mimic the periodontal ligament.

Endodontic access was made, apical patency was verified by inserting a size 10 K-file instrument (SybronEndo, Orange, CA, USA) to the apical foramen, and then the working length was set 0.5 mm short of the foramen. A glide path was made manually with K-file instruments, sizes 10 and 15. Then, root canals were prepared with HyFlex CM rotary file instruments (Coltène Whaledent, Altstätten, Switzerland) to size 30, 04. During preparation, patency and irrigation with 2.5% sodium hypochlorite (NaOCl) were performed. Finally, the canal was irrigated with 17% ethylenediamine tetraacetic acid (EDTA) and flushed with normal saline. The irrigants were delivered using side-vented needles (30 Gauge).

The prepared teeth were randomly coded (using www.random.org accessed on 9 December 2020) and distributed equally into 2 groups, according to which endodontic sealer was used, namely AHP and TFBC sealers. After drying the canal, the tested sealer was applied inside the canal and at the apical 5 mm of the gutta-percha master cone (size 30, 04 taper). The coated cone was then inserted to the working length provided that the tug-back sensation was achieved. The excess gutta-percha was cut just apical to the cementoenamel junction level by using the B&L SuperEndo Alpha II unit (B&L BioTech, Philadelphia, PA, USA). The obturation was performed by a single operator. The access cavity was temporized with Coltosol F (Coltène Whaledent, Altstätten, Switzerland) and a periapical radiograph was taken to confirm the obturation quality. Then, each tooth was placed in a test tube filled with phosphate-buffered saline (PBS) and incubated at 37 °C and 100% humidity for 6 months.

### 2.3. Root Canal Retreatment

The retreatment procedure was performed by an experienced endodontist (A.J.) who was not aware of the type of sealer used in each tooth. The teeth were decoronated with a diamond disc to a level that left a working length of 14 mm. Because the performance of the XP Shaper (FKG Dentaire SA, La Chaux-de-Fonds, Switzerland) is affected by the ambient temperature, and in order to mimic the clinical conditions, the experiment was conducted by surrounding the tooth root with warm water (35 [±1] °C) during the investigation.

Gates Glidden drills, size 3 (Densply Sirona, Ballaigues, Switzerland), were used to remove the coronal 2 mm of obturating materials. A few drops of chloroform were placed in the created coronal space and left for 1 min to soften the obturating material and facilitate instrument penetration. Under a dental operating microscope (4× magnification), an XP Shaper instrument (size 27, 0.01 taper) was operated at a 3000 rpm speed and MAX torque using a digital torque control motor (SybronEndo, Orange, CA, USA) to prepare through the gutta-percha. Efforts were made to reach the working length. Then, 10 vertical strokes with a brushing motion were performed. Afterward, the canals were dried with paper points and inspected for any remnants of gutta-percha at 8× magnification. If any gutta-percha was visible on the root canal walls, another 10 vertical strokes were applied. The canals were irrigated with 2.5% NaOCl throughout the procedure and a final rinse of 17% EDTA before flushing with normal saline, followed by drying with paper points. The effective retreatment time, which exhibited the active retreatment steps with the XP Shaper instrument, was calculated, and regaining the apical patency was checked using a size 15 K-file. Then, all teeth roots were placed in the PBS and stored back in the incubator until further use.

### 2.4. Vertical Force and Torque Measurements

Prior to the retreatment procedure, the force gauge was secured and centered in a standing position above the torque gauge. Then, the tooth root was firmly fixed in the center at the top of the force gauge and the accuracy of the 2 gauges was ensured. With this assembly, the developed real-time force and torque were measured simultaneously using MESUR Lite software (Mark-10 Corporation, Copiague, NY, USA). The accuracy of the gauge devices was ensured. The forces were shown in 2 directions: apically and coronally. The apically directed force represents the positive force needed to insert the instrument into the canal space. The coronally directed force represents the force developed when the instrument was withdrawn from the canal space against friction. The positive torque values were recorded. The gauge devices measured data every 0.1 s.

### 2.5. Micro-Computed Tomography (Micro-CT) Evaluation

The roots were imaged using a micro-CT device (SkyScan 1172; Bruker micro-CT, Kontich, Belgium) before and after the retreatment procedure. For consistency and fair comparison, the settings were kept the same for each scan: 70 kV, 139 μA, 0.5 mm-thick aluminum filter, 13.6 μm voxel size, 0.80 degree rotational step, 180 degree rotational angle, 2.0 s exposure time, and 3× frame averaging.

The resultant JPG slice images were used for three-dimensional reconstruction with NRecon 1.7.1.0 software (Bruker micro-CT) using 10× ring artifact correction, 50% beam hardening correction, and minimum and maximum contrast limits. Segmentation and thresholding procedures inside the region of interest (apical 12 mm) were performed using CTAn software (Bruker micro-CT) to measure the volumes of both obturating materials before and after retreatment.

Data from apical, middle, and coronal thirds (4 mm each) were investigated separately and overall. The volume percentage of the remaining obturating material was calculated by dividing the volume of the remaining obturating material (2nd scan) by the volume of the original obturating material (1st scan) and multiplying the result by 100.

### 2.6. Statistical Analysis

Statistical analyses were performed using the statistical program SPSS version 22 (IBM, Chicago, IL, USA) at a 5% significance level. Due to the absence of data normality as tested by the Shapiro–Wilk test (*p* < 0.05), the Mann–Whitney U test was employed to compare the experimental groups in terms of the effective retreatment time, the maximum vertical force in apical and coronal directions, the maximum torque, and the percentage of remaining obturating materials. The number of teeth where apical patency was regained in both groups was compared by the chi-square test.

## 3. Results

The mean and standard deviation data of the effective retreatment time, maximum retreatment forces, and torques for AHP and TFBC groups are presented in Table 1. 

TFBC required less retreatment time than AHP; however, this did not reach the level of significance (44.38 (±13.73) versus 53.93 (±23.34) s) (*p* = 0.418). In both groups, the apically and coronally directed maximum forces were in the ranges of 1.54–2.62 N force and 0.67–0.73 N force, respectively. The attained maximum torque ranged from 0.74 to 1.13 Ncm. The developed apically directed maximum force in AHP was higher than that in TFBC (*p* = 0.003). The coronally directed maximum force and the maximum torque were comparable between the groups (*p* = 0.223). The apical patency was reestablished in all TFBC roots and only 69% of the AHP roots (chi-square test; *p* = 0.030).

**Figure 1:**
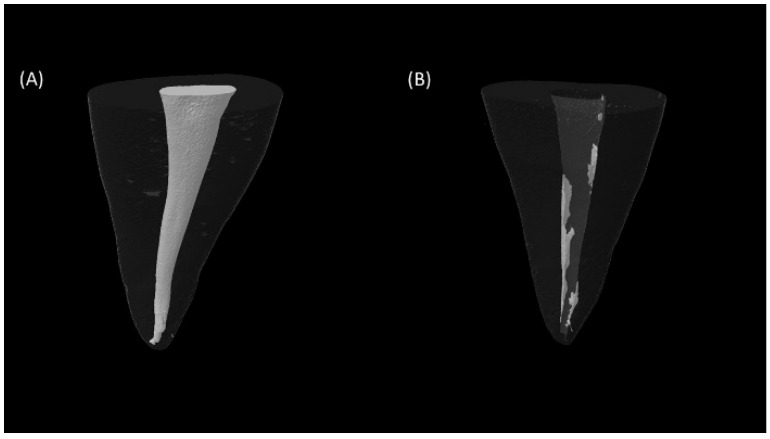
Reconstructed 3D microCT images for a representative sample. (**A**) Root canal obturating materials before retreatment and (**B**) the remaining obturating materials after retreatment.

**Figure 2:**
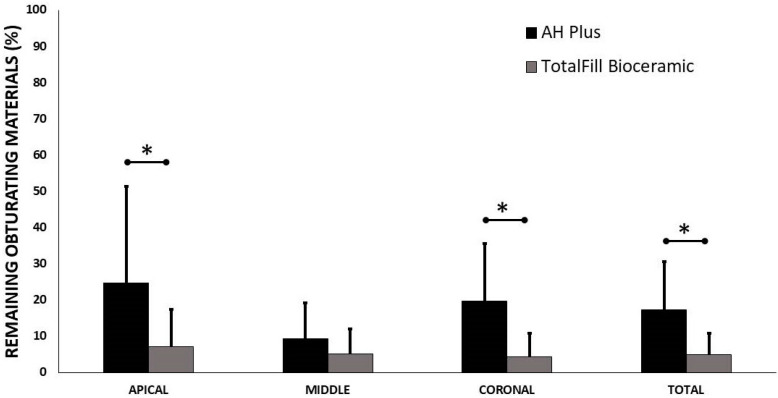
Mean percentages of the remaining obturating materials on the overall root canal and on each root canal third (coronal, middle, or apical third) after retreatment of the experimental groups. The asterisk represents statistical significance.

Micro-CT 3D images before and after retreatment of a representative sample of each group are shown in Figure 1. The mean percentages of the remaining obturating materials on the overall root canal surface and on each root canal surface third (coronal, middle, or apical) are shown in Figure 2. AHP-containing roots had higher amounts of remaining obturating materials overall, while the coronal and apical root canal surfaces were comparable to those that had TFBC (*p* = 0.007).

## 4. Discussion

The present findings provide evidence on how some root canal retreatment parameters are considerably affected by the type of endodontic sealer used during the initial treatment. TFBC-obturated roots exhibited lower apically directed forces and a lesser amount of remaining obturating materials along with regaining apical patency in all roots. However, the retreatment time, the coronally directed force, and the maximum torque were not statistically different between groups. Thus, the null hypothesis was rejected.

To the authors’ knowledge, this is the first study to investigate the force and torque induced during root canal retreatment. The XP Shaper instrument generated low forces and torques during initial treatment [21]. In the present study, higher apical forces (>40%) and torques (>30%) were observed, although the rotational speed used for retreatment was three times higher than the speed used for initial treatment. It was speculated that the impediment imposed by the obturating materials results in more stress on the root canal walls that is then translated into the application of higher force and torque. The type of obturating materials used has an influence on the generated force, as seen in the present study, where roots obturated with AHP showed higher apically directed forces when compared with those obturated with TFBC. Moreover, although it is not significant, TFBC-obturated roots required less retreatment time than AHP-obturated roots, which corroborates with a previous finding [12].

Clinically, what appears as a successful root canal treatment may fail at a later time. This might be attributed to the low quality of the root canal obturation and/or postendodontic restorations [3]. Hence, there is always a need to test the retreatability of obturated canals, especially with the frequent introduction of new materials into the market and taking into consideration the changes that might happen to the properties of root-filling material over time. Indeed, some characteristics of the obturating materials inside root canals have been affected with storage [26,27,28,29]. Besides that, a recent study revealed that the voids’ volume increased in root canals obturated with calcium silicate-based sealers after 2 months [30]. These alterations may ease or hamper their removal during retreatment. Therefore, in the present study, the tested teeth were aged for 6 months before commencing retreatment in order to simulate the clinical scenario. 

The XP Shaper has a snake shape with a triangular cross-section, which is claimed to allow the instrument to prepare the root canal system in three dimensions [31], with less stress imposed on the canal walls [21]. This unique design accompanied by high-speed rotation is reported to be of great benefit during retreatment [31]. However, the instrument was unable to completely remove the obturating materials, and this is in line with the general agreement that there is no technique that fully eliminates all materials from the root canal system [9,10,11,12,13,14,15,16,17,18,19]. The remaining materials were mostly located in the buccal and lingual walls. This might be due to the inclusion of oval canals, which added a challenge to the complete removal of obturating materials even with the use of the XP Shaper instrument, which is considered to be efficient in retreatment at high speed and is designed to contact a great area of the canal walls [32]. TFBC and AHP were able to remove 95% and 82.8% of the obturating materials, respectively. This finding is similar to published studies which showed that bioceramic sealers might be easily peeled off from root canals [12,13,14,15,16,19]. Hence, this gives the indication of easier and more predictable retreatment in roots obturated with TFBC when compared with AHP under the circumstances used in the present study. The low amount of remaining obturating materials along with the low retreatment forces and torques observed with TFBC might be attributed to its bond strength to dentin and solubility. Inferior push-out bond strength was found in teeth with bioceramic sealer compared to teeth with AHP [33,34,35]. It was stated that the bonding mechanism of bioceramic sealers to root dentin after setting is not adequately addressed [36]. The solubility characteristics of an endodontic sealer are another integral property since its dissolution may compromise the overall quality of the root canal treatment and the ability to prevent apical leakage. A meta-analysis revealed an overall higher solubility of TFBC compared to the AHP as a result of the unpredictable setting of bioceramic sealer and hydrophilic particles that allow for the absorption of more liquid over time [37]. This warrants further research attention to achieve a better understating of the characteristics of bioceramic sealers.

Achieving canal patency with effective cleaning close to the canal exit has been recognized as a prognostic factor for the successful healing of periapical tissue [38]. In this study, the apical patency was regained in all TFBC teeth and only 69% of the AH Plus teeth. Past studies have reported that apical patency was regained in 100% of samples with bioceramic sealers [13,18]. Another previous study found that 80% of the bioceramic samples regained the patency [10]. These findings might indicate that retreatment of bioceramic sealers can be accomplished.

Although apical patency was regained in most of the tested teeth, the apical third was found to harbor a great amount of remaining obturating materials in both groups. This is consistent with other studies [9,11,15,17], which are greatly influenced by the anatomic complexity in the apical third.

In this study, there are certain limitations. The utilization of extracted teeth from different human subjects and the use of in vitro experimental conditions can provide only limited answers to complex problems. Therefore, great care was taken to balance all potential variables in order to purely investigate the influence of endodontic sealers on retreatment. All the tested teeth had single oval canals and were decoronated prior to retreatment to standardize the working length to 14 mm. Then, the teeth were balanced in both groups concerning the overall volume of obturating materials (*p* = 0.248). The observation volume was limited to the apical 12 mm (4 mm for each third) to avoid the coronal area prepared by the Gates Glidden drill. The retreatment procedure was performed in strokes with gentle in-and-out movements to keep consistent pressure on the instrument where the endodontist was blinded to the type of sealer present in each tooth.

Caution should be taken when interpreting the study findings. The current experiment was performed on oval canals obturated using matched gutta-percha cone technique. Although the sealer filled most of the tested canals, the results showed that the removal of obturating materials in AHP was more difficult than that in the TFBC.

This study focused on two types of sealers removed by one brand of NiTi instruments. Thus, further investigations should be performed with other NiTi instrument systems dedicated for retreatment with different endodontic sealers where differences in the endodontic sealer and the tested instrument might exhibit different remaining obturation as well as developed force and torque during the retreatment.

## 5. Conclusions

Under the conditions of this study, endodontic sealer had an influence on the amount of remaining obturating materials and developed force where the TFBC showed less remaining materials and lower retreatment forces in the apical direction when compared with the AHP in extracted teeth with oval canals.

## Figures and Tables

**Table 1:** Descriptive data (mean ± standard deviation) of the effective time, maximum retreatment forces, and torques induced in each group.

GROUP	Effective Time (s)	Maximum Force (N)		Maximum Torque (Ncm)
		Apically Directed	Coronally Directed	
AH Plus	53.93 ± 23.34	2.62 ± 1.20	0.67 ± 0.41	1.13 ± 1.04
TotalFill	44.38 ± 13.73	1.54 ± 0.61	0.73 ± 0.32	0.74 ± 0.51
*p* value (Mann–Whitney test)	0.418	0.003	0.223	0.479

## Data Availability

All the data are contained within the article.
